# Polycaprolactone-Modified Biochar Supported Nanoscale Zero-Valent Iron Coupling with *Shewanella putrefaciens* CN32 for 1,1,1-Trichloroethane Removal from Simulated Groundwater: Synthesis, Optimization, and Mechanism

**DOI:** 10.3390/molecules28073145

**Published:** 2023-03-31

**Authors:** Jing Ye, Yacen Mao, Liang Meng, Junjie Li, Xilin Li, Lishan Xiao, Ying Zhang, Fenghua Wang, Huan Deng

**Affiliations:** 1 School of Chemical and Environmental Engineering, Shanghai Institute of Technology, Shanghai 201418, China; 2 Key Lab of Eco-Restoration of Regional Contaminated Environment, Ministry of Education, Shenyang University, Shenyang 110044, China; 3 School of Environmental and Geographical Sciences, Shanghai Normal University, Shanghai 200234, China; 4 Yangtze River Delta Urban Wetland Ecosystem National Field Scientific Observation and Research Station, Shanghai 201722, China; 5 State Environmental Protection Key Laboratory of Synergetic Control and Joint Remediation for Soil & Water Pollution, Chengdu University of Technology, Chengdu 610059, China; 6 The Shanghai Key Lab for Urban Ecological Processes and Eco-Restoration, East China Normal University, Shanghai 200241, China; 7 School of Geographical Sciences, Hebei Normal University, Shijiazhuang 050024, China; 8 School of Environment, Nanjing Normal University, Nanjing 210023, China

**Keywords:** polycaprolactone-modified biochar, nanoscale zero-valent iron, dissimilatory iron reduction, 1,1,1-trichloroethane, groundwater remediation

## Abstract

1,1,1-Trichloroethane (1,1,1-TCA) is a typical organochloride solvent in groundwater that poses threats to human health and the environment due to its carcinogenesis and bioaccumulation. In this study, a novel composite with nanoscale zero-valent iron (nZVI) supported by polycaprolac-tone (PCL)-modified biochar (nZVI@PBC) was synthesized via solution intercalation and liquid-phase reduction to address the 1,1,1-TCA pollution problem in groundwater. The synergy effect and improvement mechanism of 1,1,1-TCA removal from simulated groundwater in the presence of nZVI@PBC coupling with *Shewanella putrefaciens* CN32 were investigated. The results were as follows: (1) The composite surface was rough and porous, and PCL and nZVI were loaded uniformly onto the biochar surface as micro-particles and nanoparticles, respectively; (2) the optimal mass ratio of PCL, biochar, and nZVI was 1:7:2, and the optimal composite dosage was 1.0% (w/v); (3) under the optimal conditions, nZVI@PBC + CN32 exhibited excellent removal performance for 1,1,1-TCA, with a removal rate of 82.98% within 360 h, while the maximum removal rate was only 41.44% in the nZVI + CN32 treatment; (4) the abundance of CN32 and the concentration of adsorbed Fe(II) in the nZVI@PBC + CN32 treatment were significantly higher than that in control treatments, while the total organic carbon (TOC) concentration first increased and then decreased during the culture process; (5) the major improvement mechanisms include the nZVI-mediated chemical reductive dechlorination and the CN32-mediated microbial dissimilatory iron reduction. In conclusion, the nZVI@PBC composite coupling with CN32 can be a potential technique to apply for 1,1,1-TCA removal in groundwater.

## 1. Introduction

1,1,1-Trichloroethane (1,1,1-TCA) is a common organochloride solvent, and has been the most frequently detected contaminant in groundwater from numerous industries (e.g., chemical, electronic, pharmaceutical, and dyeing) around the world due to its extensive usage and improper disposal [1]. Groundwater pollution with 1,1,1-TCA has elicited much attention because of its easy migration and diffusion, recalcitrant characteristic, and potential carcinogenic and mutagenic risks in humans from long-term exposure [2]. Hence, most countries have listed 1,1,1-TCA as a priority pollutant. Meanwhile, due to the complexity and difficult resilience of the subsurface, the development of high-efficiency, environment-friendly, low-cost techniques for the removal of 1,1,1-TCA in groundwater is clearly required.

The relative anaerobic environment in groundwater is conducive to the dechlorination degradation of organochloride solvents [3]. Therefore, in situ enhanced reductive dechlorination (ERD) has become a widely used technology for the remediation of groundwater contaminated by 1,1,1-TCA, which achieves desirable performance by facilitating both chemical and biological dechlorination pathways. The remedial material is a key factor that influences the treatment effect of this technology [4]. Nanoscale zero-valent iron (nZVI) is the most commonly used chemical reductant for 1,1,1-TCA dechlorination due to its large specific surface area, high reaction activity, and less secondary pollution. The major reduction mechanisms of nZVI include direct electron transfer from iron to chlorinated solvents, reduction with ferrous iron, and electrocatalytic reduction with hydrogen [5]. However, nZVI particles in an aqueous environment can easily agglomerate due to the electrostatic interaction, thus decreasing its reaction rate and migration distance in the subsurface. Moreover, organic carbon source has been considered as a most promising biological reductant for 1,1,1-TCA dechlorination. It can be used as an electron donor and energy source to improve the biological reductive dechlorination efficiency by promoting the growth of organohalide-respiring or extracellular-respiring bacteria [6]. At present, liquid organic substances are used extensively. For example, methanol, ethanol, acetic acid, and glucose are often injected into organic carbon-limited groundwater. Although these carbon sources have good carbon supply efficiency, they still have some limitations, such as fast carbon release rate, insufficient dose or overdosing risk, and rather sophisticated and costly control process [7]. Therefore, the preparation of high-efficiency functional composites is a novel approach to solving the above problems.

Biochar (BC) can be used as a mechanical supporter to enhance the stabilization and dispersivity of nanoparticles due to its large surface area, high stability, and rich porous structure, thus improving the nZVI reactivity [8]. As a porous material, it can further facilitate the reduction rate of chlorinated hydrocarbons such as 1,1,1-TCA owning to increase the contact degree between the contaminant and BC-loaded nZVI through adsorption [9]. Recent studies also found that electroactive quinoid groups and π-π conjugated structures in BC can accelerate the migration of electrons from nZVI to chlorinated hydrocarbons, thus further improving the reducing capacity of nZVI [10]. However, the relatively low bioavailable carbon content of BC cannot provide sufficient carbon sources to simulate the activity of functional bacteria responsible for reductive dechlorination [11]. Polycaprolactone (PCL) is a solid organic substance with excellent biodegradability and biocompatibility. It will continuously provide soluble organic carbons along with its decomposition by microbial extracellular hydrolase [12]. This process is not only simple and low-cost, but also can avoid excessive or deficient release of carbon sources [13]. Currently, PCL has been utilized as a constant carbon source for bacterial growth and as an electron donor for denitrification. Chu et al. used PCL as a carbon source and increased the removal rate of total nitrogen in groundwater to 95% through the denitrification process, with the significant enrichment of denitrifying bacteria such as *Diaphorobacter* [14]. This suggests that PCL might be able to facilitate the reductive dechlorination of chlorinated hydrocarbons by heterotrophic bacteria in groundwater. Additionally, BC can also improve the dispersivity of PCL in groundwater as a supporter of TCA [15]. Hence, we hypothesized that a BC composite with nZVI and PCL could effectively remove 1,1,1-TCA from groundwater by enhancing chemical and biological reductive dechlorination. To the best of our knowledge, such a composite has not yet been reported.

On the other hand, nZVI can be easily oxidized by water/oxygen, forming iron (hydr)oxides passivation layer on its surface to limit the reactivity of nZVI against pollutants. Dissimilatory iron-reducing bacteria (DIRB) can perform anaerobic respiration by using organics as electron donors and Fe(III) (hydr)oxides as terminal electron acceptors, thereby generating Fe(II) [16]. In nature, the DIRB-mediated Fe(III) reduction often occurs under anaerobic conditions. Therefore, the iron (hydr)oxides passivation layer of aged nZVI can be decomposed using DIRB in groundwater, thus prolonging the reactivity of nZVI and improving the removal efficiency of pollutants [17]. Additionally, as mentioned above, we hypothesized that PCL could continuously provide DIRB with electrons, while BC is conducive to DIRB colonization as a porous substrate and iron reduction as an electron shuttle. However, the synergy effect of these two materials on facilitating the dissimilatory iron reduction dechlorination of 1,1,1-TCA in groundwater induced by passivated nZVI and DIRB has not yet been directly confirmed.

In this study, a novel composite of BC-supported PCL and nZVI was coupled with *Shewanella putrefaciens* CN32 (CN32) to remove 1,1,1-TCA from groundwater by chemical and biological reductive dechlorination. The CN32 strain was selected because it is a typical DIRB species known to have a remarkable respiratory capacity to reduce chlorinated hydrocarbons, Fe(III), Cr(VI), U(VI), nitrite, and other electron acceptors [18]. The major objectives of this study were to (i) synthesize a novel nZVI@PBC composite by solution intercalation and liquid-phase reduction, and characterize this composite with SEM, TEM, FTIR, and XRD; (ii) evaluate the significant factors influencing 1,1,1-TCA removal by nZVI@PBC + CN32, including the component mass ratio, composite dosage, and pollutant concentration; (iii) propose the possible mechanism of 1,1,1-TCA dechlorination in stimulated groundwater via the synergy effect of nZVI@PBC and DIRB.

## 2. Results and Discussion

### 2.1. Composite Characterization

**Figure 1:**
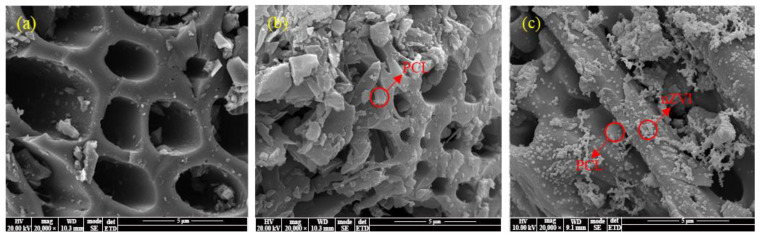
SEM images of BC (**a**), PBC (**b**), and nZVI@PBC (**c**).

Figure 1a shows that the surface of BC was relatively smooth, with abundant porous structures. Figure 1b shows that PCL adhered onto the BC surface uniformly as irregular particles (0.5~3 μm), improving the roughness of BC without covering its porous structures completely. It implies that PCL loading could further improve the adsorption capacity of the composite. It can be seen from Figure 1c that most zero-valent iron particles produced by liquid-phase reduction were also uniformly loaded on the BC surface as nanoparticles, indicating that BC could effectively prevent the nZVI aggregation. The TEM image of nZVI@PBC-20 further confirmed that nZVI particles were spherical granules with diameters of about 50~100 nm (Figure S1a). EDS mapping results suggest (Figure S1b) that C (55.09%), O (15.65%), and Fe (24.30%) were dominant elements on the composite surface. In addition, a certain O content was detected on the nZVI surface, indicating that nZVI was partially oxidized.

**Figure 2:**
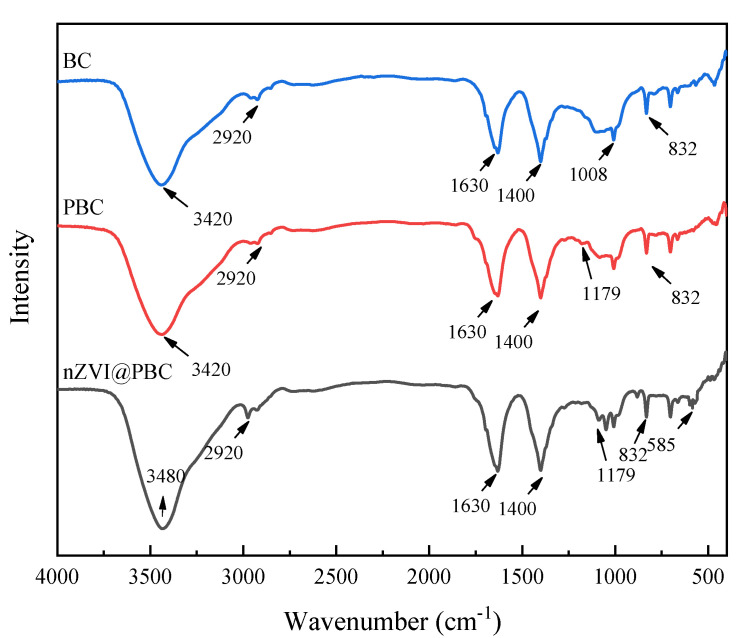
FT−IR spectra of BC, PBC, and nZVI@PBC.

The surface functional groups of BC, PBC, and nZVI/@PBC were characterized by FT-IR (Figure 2). BC exhibited abundant surface functional groups. Typically, a broad peak at approximately 3420 cm^−1^ corresponded to the O-H band, indicating that some hydroxyl groups were existed on the BC surface [19]. The peak at 2920 cm^−1^ was related to the C-H band of long-chain aliphatic components [20]. Peaks around 1630 cm^−1^ and 1400 cm^−1^ are assigned to stretching vibrations of C=C and C=O on the aromatic structures [21]. The weak bands at about 1008 cm^−1^ (C-O) and 832 cm^−1^ (C-H) were due to the phenols and furans in the aromatic rings, respectively [22]. Aside from these characteristic peaks, the FT-IR spectra of PBC showed a new peak at 1179 cm^−1^ (C=O), which can be attributed to the presence of PCL [23]. This indicates that PCL was successfully loaded onto BC. Compared to BC and PBC, a blue or red shift in most bands was observed in nZVI@PBC. For instance, the O-H band shifted from 3420 cm^−1^ for PBC and BC to 3480 cm^−1^ for nZVI@PBC, implying the bonding of nZVI with PBC [24]. Additionally, there was a new peak at 585 cm^−1^, which corresponded to Fe-O stretching, indicating that nZVI on the PBC surface was partially oxidized to form high-valent iron oxidants [25]. This is in agreement with the TEM result.

**Figure 3:**
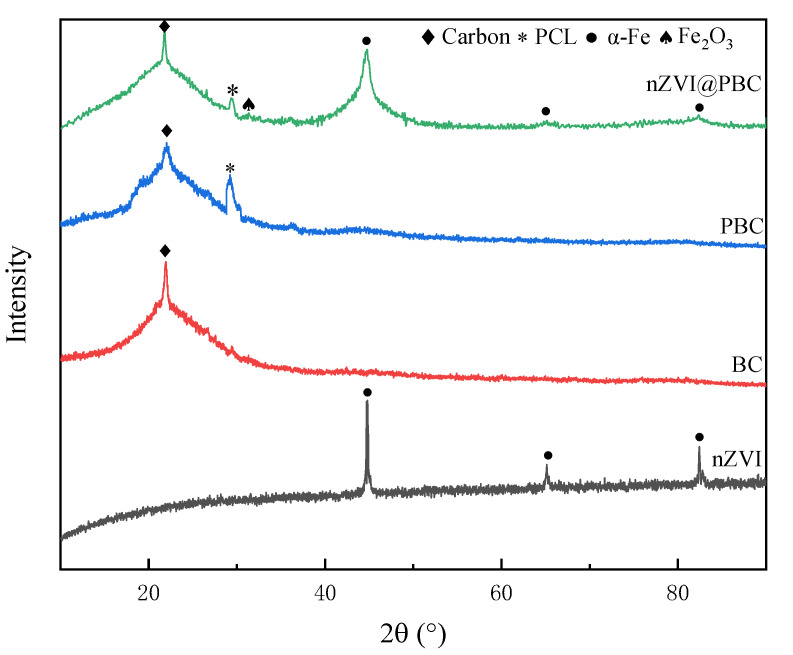
XRD patterns of nZVI, BC, PBC and nZVI@PBC.

The crystalline structures of nZVI, BC, PBC, and nZVI@PBC were evaluated by XRD with the patterns shown in Figure 3. nZVI produced through liquid-phase reduction exhibited the main diffraction peaks of α-Fe^0^ at 2*θ* = 44.8°, 64.9° and 82.3°, indicating it had a body-centered cubic structure [26]. The XRD patterns of BC and PBC both show the obvious characteristic peak of amorphous structure of carbon at 2*θ* = 23.1° [27], but PBC additionally exhibited a new characteristic peak at 2*θ* = 29.40°, which was assigned to the crystallographic plane of semi-crystalline PCL. These above characteristic peaks were also observed in nZVI@PBC, further indicating that PCL and nZVI were successfully loaded onto BC. However, the spectrum of nZVI in the composite exhibited obvious shrunk, demonstrating that the introduction of BC and PCL effectively enhanced the dispersal and uniformity of nZVI [28]. The XRD pattern of nZVI@PBC also showed a small Fe_2_O_3_ peak at 2*θ* = 33.1°, further indicating a small amount of nZVI in the composite was oxidized during synthesis. Moreover, the characteristic peak of PCL became weakened attributed to nZVI coating [29].

### 2.2. Optimization of Component Mass Fractions

#### 2.2.1. Optimal Mass Fraction of PCL

The actual mass fractions of PCL in PBC-1, PBC-2, PBC-3 and PBC-4 measured by TGA were 6.19%, 12.58%, 25.43% and 37.41% (TGA figures not show). These values are basically consistent with the theoretical ones, indicating that the PCL preparation method was reasonable.

SEM images of the four PBCs are shown in Figure S2. It can be seen that the porous structures of BC were gradually blocked with an increase in the PCL mass fraction. When the mass fraction was 37.5%, the porous structures were completely blocked. According to the BET analysis (Table S1), the specific surface area, total pore volume and pore diameter of PBC generally declined with increasing its mass fraction, but the micropore volume did not significantly change. When the mass fraction increased from 6.25% to 37.5%, the specific surface area, total pore volume and pore diameter of PBC decreased by 49.54%, 50.18% and 25.36%, respectively. To sum up, the PCL mass fraction plays an important role in the porous structural characteristics of the composite, and thus influences its adsorption capacity.

**Figure 4:**
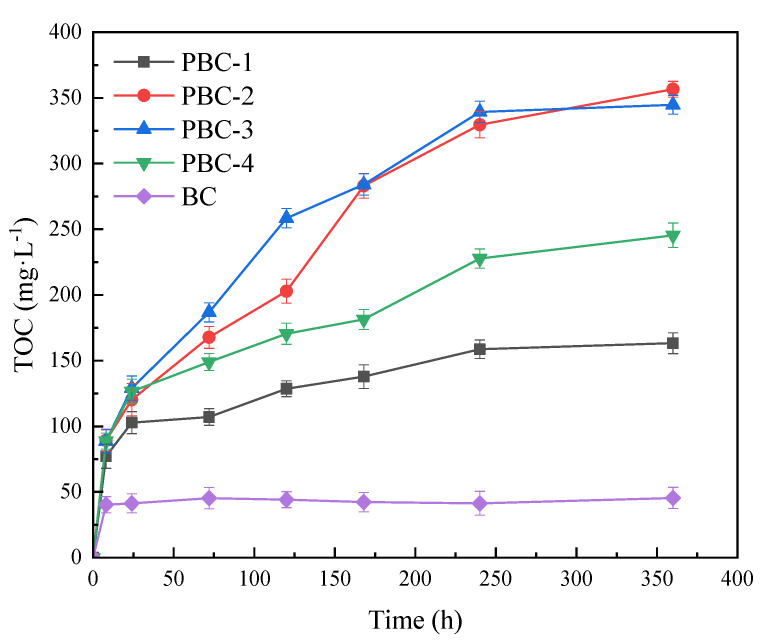
Carbon−releasing performances of BC and PBCs in sterile pure water.

The carbon-releasing performances of the four PBCs in sterile pure water are shown in Figure 4. Compared to BC, the TOC concentration in the water with PBCs increased slowly and continuously, being consistent with the carbon-releasing trend of the PCL-peanut shell carbon composite produced by Xiong et al. [6]. This indicates that PCL loading could improve the slow carbon releasing performance of the composite. After 360 h, the TOC concentrations in PBC-1, PBC-2, PBC-3 and PBC-4 treatments were 161.33, 297.61, 281.56 and 243.35 mg·L^−1^, respectively. The carbon amount released from PBC-4 with the highest PCL content was relatively lower compared to those released from PBC-2 and PBC-3. This might be because the excessive PCL would result in agglomeration on the BC surface, thus inhibiting the carbon-releasing efficiency of PBC. Hence, with comprehensive consideration of the SEM, BET and carbon releasing experiment results, the optimal mass fraction of PCL in PBC was determined to be 12.5%.

#### 2.2.2. Optimal Mass Fraction of nZVI

The mass fractions of nZVI in nZVI@PBC-5, nZVI@PBC-10, nZVI@PBC-20 and nZVI@PBC-30 measured by ICP-MS were 4.38%, 10.21%, 20.75% and 29.63%, respectively. These values are basically consistent with the theoretical ones, further indicating that nZVI was successfully loaded onto BC surface by liquid-phase reduction.

The BET results of the four composites are shown in Table 1. Generally speaking, the specific surface area of the composite increased with increasing the nZVI mass fraction. However, the specific surface area of nZVI@PBC-30 with the highest nZVI content was less than those of other three composites. The reason is that excessive nZVI would be easy to form aggregates and block the pore structure of BC, thus inducing the decline of the composite specific surface area. Additionally, the total pore volume and micropore volume of the composites were negatively related to the nZVI mass fraction. The FT-IT results (Figure S3) show that the nZVI mass fraction did not significantly affect characteristic peaks of BC and PCL, but the Fe-O peak became stronger with increasing nZVI content, indicating that the excessive nZVI were more likely to be oxidized.

**Figure 5:**
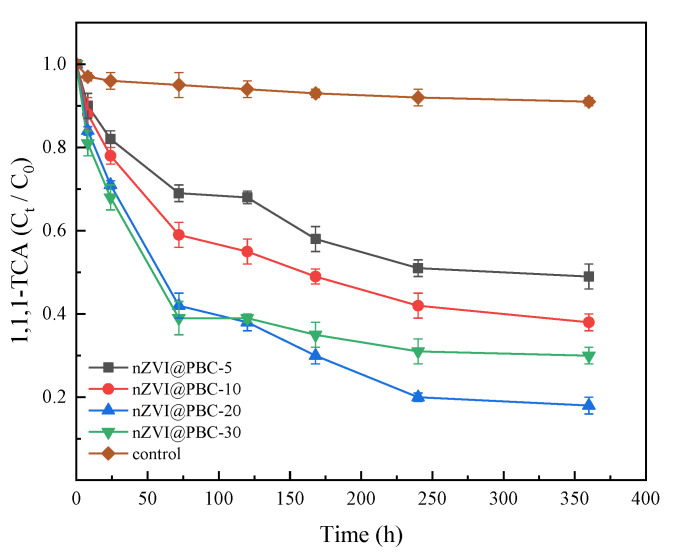
1,1,1-TCA removal performances of composite materials with different nZVI contents coupling with CN32 (*C*_0_: initial 1,1, 1-TCA concentration; *C*_t_: 1,1, 1-TCA concentration at time t; composite dosage: 1.00% *w/v*; 1,1,1-TCA concentration: 100 mg·L^−1^).

The synergy effects of the four composites and *Shewanella putrefaciens* CN32 on the removal of 1,1,1-TCA (100 mg·L^−1^) from simulated groundwater are shown in Figure 5. At the end of the culture period, the removal rate of 1,1,1-TCA in the different composite treatments (58.79–75.27%) was significantly higher than that in the control group without composite addition (12.3%). This indicates that the composites produced in this study had good performance on 1,1,1-TCA removal from groundwater. Specifically, the 1,1,1-TCA concentration in the composite treatments declined obviously within 72 h, and the declined degree increased with increasing nZVI mass fraction. This is because that nZVI can achieve rapid dichlorination of 1,1,1-TCA in a short period due to its strong chemical reduction ability, and the reduction rate is proportional to the nZVI dosage [30]. After 72 h, the decline of 1,1,1-TCA concentration in the composite treatments slowed down. The reason is considered that nZVI has been almost depleted in the early stage of the reaction, and thus the 1,1,1-TCA removal is predominantly induced by the CN32-mediated reductive dichlorination [31]. Meanwhile, the residual concentration of 1,1,1-TCA in nZVI@PBC-20 treatment after 72 h was obviously lower than those in the other composite treatments. Because of the low nZVI content in nZVI@PBC-5 and nZVI@PBC-10, the chemical reductive dechlorination was relatively weak in the two treatments, thus resulting in high residual concentrations of 1,1,1-TCA in the early reaction stage. This subsequently had toxic effect on CN32, inhibiting its activity of dissimilatory iron reduction. On the other hand, although nZVI content was the highest in nZVI@PBC-30, the BC and PCL contents were relatively low compared to those in other composites, thus weakening its stimulation on CN32 growth. Furthermore, the agglomeration of excessive nZVI in nZVI@PBC-30 might also decrease its chemical reductive dechlorination capacity [32].

To sum up, it appears that the mass fraction of nZVI in nZVI@PBC-20 was optimal. Therefore, the optimal mass fractions of BC, PCL and nZVI in the composite were 70%, 10% and 20%, respectively. Accordingly, the optimal mass ratio of PCL, BC and nZVI was 1:7:2. “Composite” or “nZVI@PBC” mentioned in follow-up experiments refers to nZVI@PBC-20.

### 2.3. Effects of Composite Dosage on 1,1,1-TCA Removal

**Figure 6:**
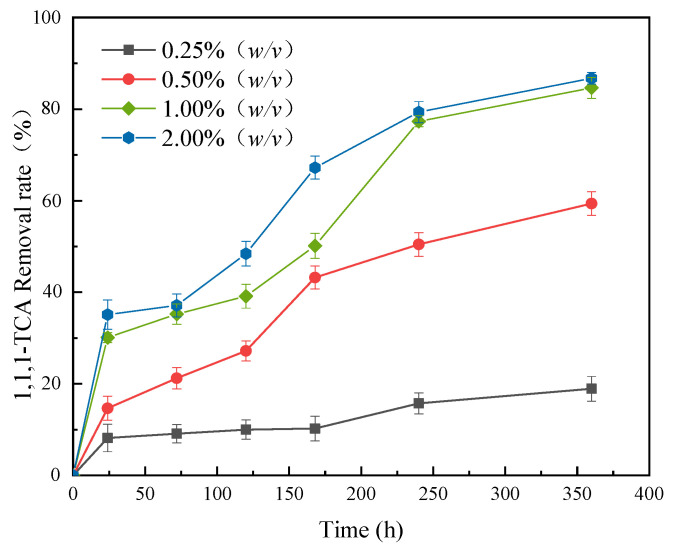
Effect of composite dosage on 1,1,1-TCA removal (mass fraction of nZVI: 20%; 1,1,1-TCA concentration: 100 mg·L^−1^).

The synergy effects of the composites with different dosages coupling with CN32 on 1,1,1-TCA removal from simulated groundwater are shown in Figure 6. The removal rate of 1,1,1-TCA increased with increasing composite dosage. When dosage increased from 0.25% (*w/v*) to 2.00% (*w/v*), the removal rate of 1,1,1-TCA in the simulated groundwater increased from 18.92% to 86.73% after 360 h. As mentioned above, this is because the chemical reductive dechlorination of nZVI and the stimulation of BC and PCL on the activity of CN32 were relatively weak when the composite dosage was less. However, the increasing degree of removal rate slowed down when the composite dosage exceeded 1.00% (*w/v*), indicating that the pollutant removal capacity of the composite tended to be stable with further increasing the dosage. Taking 1,1,1-TCA removal and preparation costs into account, the optimal composite dosage was determined to be 1.00% (*w/v*).

### 2.4. Effects of 1,1,1-TCA Concentration on 1,1,1-TCA Removal

**Figure 7:**
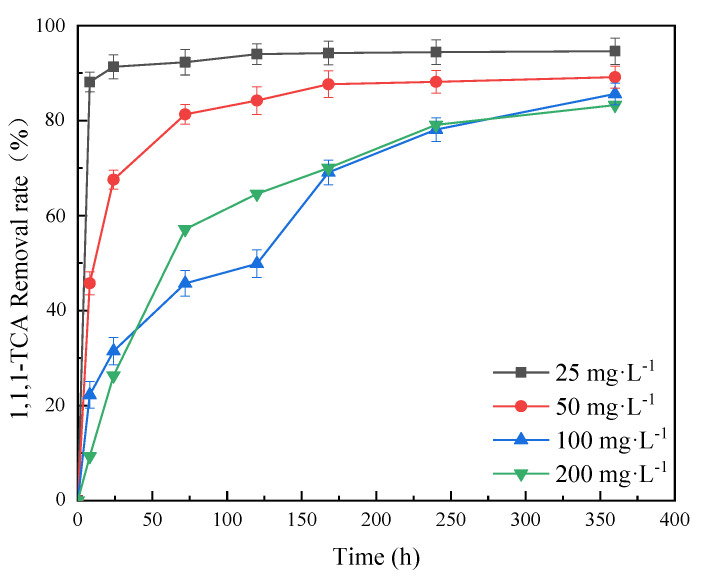
Effects of 1,1,1−TCA concentration on 1,1,1−TCA removal (mass fraction of nZVI: 20%; composite dosage: 1.00% *w/v*).

The synergy effects of nZVI@PBC (dosage: 1.00% *w/v*) and CN32 on the removal of 1,1,1-TCA at different concentrations from simulated groundwater were further investigated (Figure 7). The results demonstrate that the removal rate of 1,1,1-TCA decreased with increasing 1,1,1-TCA concentration to some extent. When the 1,1,1-TCA concentrations were 25, 50, 100 and 200 mg·L^−1^, the removal rates after 360 h reached 94.61%, 89.16%, 85.62% and 83.27%, respectively. This indicates that the composite with optimal component mass ratio and dosage coupling with CN32 could effectively remove 1,1,1-TCA at virous concentrations, implying it has a good application potential for the remediation of groundwater with different chlorinated hydrocarbon pollution levels. Bae et al. proved that 1,1,1-TCA is a strong oxidant as well as a passivator for nZVI [33]. When the initial concentration was relatively high, 1,1,1-TCA could strongly react with nZVI to rapidly form a compact passivation layer of Fe(III) oxides/hydroxides on BC surface, thus hindering the chemical reductive dichlorination by the un-oxidized nZVI. The SEM image of nZVI@PBC-20 after 72 h of reaction also shows an obvious iron oxide passivation layer developed on the composite surface (Figure S4). However, the removal rate of 1,1,1-TCA at high concentration increased constantly during the whole incubation period in this study. This might be because CN32 could reduce Fe(III) in the passivation layer into Fe(II) through microbial dissimilatory iron reduction, thus maintaining the reductive dechlorination capacity of the composite to high-concentration 1,1,1-TCA [34].

### 2.5. Mechanism of 1,1,1-TCA Removal by nZVI@PBC Coupling with CN32

**Figure 8:**
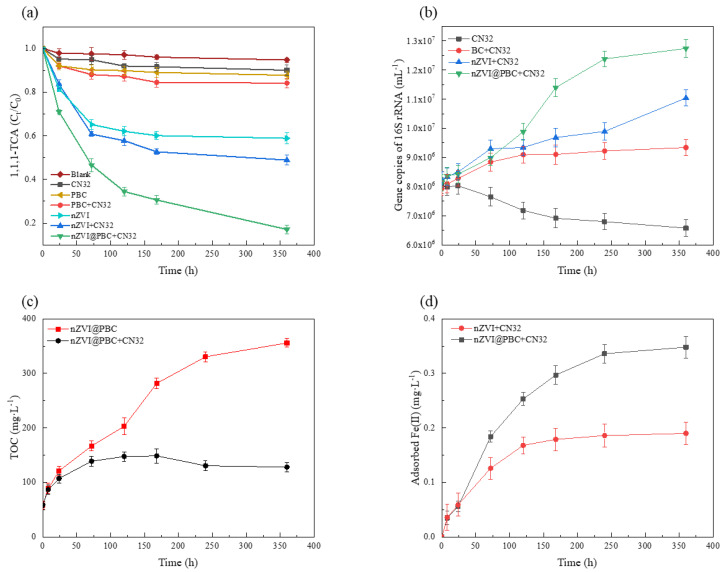
1,1,1−TCA removal performances (**a**), 16S rRNA copy numbers of CN32 (**b**), TOC concentrations (**c**) and adsorbed Fe(II) concentrations (**d**) in nZVI@PBC + CN32 and other control treatments over time.

To preliminarily explore the mechanism of 1,1,1-TCA removal by the composite coupling with CN32, variations in 1,1,1-TCA, TOC and adsorbed Fe(II) concentrations, and 16S rRNA copy numbers of CN32 in nZVI@PBC + CN32 and other control treatments over time were analysed (Figure 8). The dosage of nZVI@PBC was 1.00% (*w/v*). The dosages of PBC and nZVI in other control treatments were consistent with their contents in nZVI@PBC + CN32 treatment. The initial concentration of 1,1,1-TCA in all treatments was 100 mg·L^−1^.

It can be seen from Figure 8a that nZVI@PBC + CN32 treatment achieved the highest 1,1,1-TCA removal rate (82.98%) after 360 h, while those in PBC and nZVI treatments were 12.29% and 41.11%, respectively. This suggests that the physical adsorption of BC and the chemical reduction directly mediated by ZVI were not the major pathways for 1,1,1-TCA removal by the composite coupling with CN32. The microbial dissimilatory iron reduction might play an important role on 1,1,1-TCA removal, which could be induced by DIRB depleting the Fe(III) (hydr)oxides passivation layer formed on the composite surface [35]. Meanwhile, the 1,1,1-TCA concentration in nZVI@PBC + CN32 treatment decreased continuously throughout the incubation period, while the 1,1,1-TCA removal rate in nZVI + CN32 treatment had been already stable at 168 h (47.30%). This demonstrates that nZVI@PBC had a good long-term effectiveness in the present of CN32.

Figure 8b shows that the 16S rRNA copy number of CN32 increased continuously over time in treatments amended with PBC, nZVI and nZVI@PBC as compared to CN32 treatment, and the greatest increase was observed in nZVI@PBC + CN32 treatment after 360 h. This is because the abundant porous structures in BC could provide suitable habitats for CN32 growth [36]. It is also attributed to the rapid decline of 1,1,1-TCA concentration by nZVI reduction in a short time, thus minimizing the 1,1,1-TCA toxicity to CN32 [37] As shown in Figure 8c, the TOC concentration in nZVI@PBC treatment increased slowly over time, while that in nZVI@PBC + CN32 treatment first increased and then decreased. The above results indicate that the composite also had a slow carbon releasing performance in the simulated groundwater, and the released organic carbons could be fully utilized by CN32 as energy sources and electron donors [38]. Therefore, it can be considered that the stimulation of DIRB growth by the slow-releasing carbon sources was important to ensure the high-efficiency and long-term performance of the composite on 1,1,1-TCA removal in the groundwater.

It has been proved that Fe(II), generated from the dissolution of the Fe(III) (hydr)oxides passivation layer through DIRB-mediated dissimilatory iron reduction, mainly exists in an adsorbed state on the biochar surface, which has more effective reducing capacity than dissolved Fe(II) and nZVI [39]. Figure 8d shows that the adsorbed Fe(II) concentrations in nZVI@PBC + CN32 and nZVI + CN32 treatments both increased continuously over time. Moreover, the 1,1,1-TCA removal rates and CN32 abundances in the two treatments were relatively high (Figure 8a,b). Therefore, it seems that the passivation layer on the composite surface, generated during the nZVI-induced chemical reductive dichlorination, could be dissolved and form adsorbed Fe(II) through the dissimilatory iron reduction mediated by CN32, thus improving the 1,1,1-TCA removal efficiency in the present of nZVI@PBC coupling with CN32. Li et al. [17] also found that adsorbed Fe(II) formed on iron minerals could enhance the dechlorination ability of DIRB to degrade pentachlorophenol. Additionally, BC might accelerate the transfer of electrons between CN32 and adsorbed Fe(II) as a solid-phase electron shuttle, which could also facilitate the dissimilatory iron reduction dichlorination occurred in this synergy system [40].

**Figure 9:**
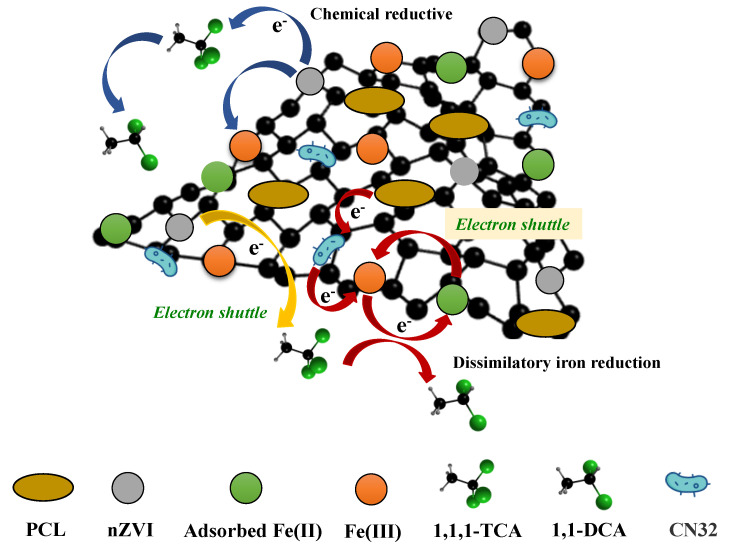
The mechanism scheme for 1,1,1-TCA removal enhanced by nZVI@PBC coupling with CN32.

According to the above results, it can be concluded that the mechanism of 1,1,1-TCA removal enhanced by nZVI@PBC coupling with CN32 was a comprehensive result of the synergism of components in the composite (Figure 9). In the early stage, the rapid chemical reductive dichlorination induced by nZVI is the main pathway to remove 1,1,1-TCA. After the depletion or oxidation of nZVI, the CN32 growth is promoted by BC as a colonization supporter and PCL as a slow-releasing carbon source, meanwhile, the electron transfer in the synergy system is also accelerated by BC as an electron shuttle. These can obviously stimulate the pathway of dissimilatory iron reduction mediated by CN32, thus facilitating the dissolution of the Fe(III) (hydr)oxides passivation layer on the surface of nZVI@PBC and extending its reactivity by the formation of adsorbed Fe(II) as a strong reductant. The above synergism finally achieves the high-efficiency and long-term removal of 1,1,1-TCA by the studied composite.

## 3. Material and Methods

### 3.1. Materials

1,1,1-TCA (99.9%) was purchased from Sigma Aldrich (USA). Polycaprolactone ([C_4_H_6_O_2_]_n_) with a molecular weight of 50 kDa was purchased from Daigang Biology Co., Ltd. (Jinan, China). Piperazine-1,4-bisethanesulfonic acid (PIPES), FeCl_2_·4H_2_O, NaBH_4,_ and other chemicals were purchased from Sinopharm Chemical Reagent Co., Ltd. (Shanghai, China). All chemicals were of analytical grade or above and were used without any further purification. All solutions were prepared using 18 MΩ deionized water (Milli-Q Gradient, Millipore, MA, USA).

### 3.2. Modification of BC with PCL

BC was produced by the pyrolysis of reed straw as the raw biomass material. The reed straw was collected from Jinshan District, Shanghai, China. The specific preparation method and physiochemical properties of the reed straw BC have been described previously [41]. Before use, the BC passing through the 120-mesh sieve was washed with 1.2 mol·L^−1^ HCl by stirring overnight, rinsed to neutral with deionized water, and then oven dried at 80 °C. PCL-modified BC (PBC) was prepared by the solution intercalation method: 0.5 g of PCL and 7 g of BC were dispersed in 250 mL of dichloromethane by stirring for 8 h at ambient temperature. Then, the mixture was centrifuged at 8500 rpm for 5 min. The solid phase was rinsed with anhydrous ethanol and deionized water several times, and then vacuum dried at 50 °C to obtain PBC. The theoretical mass fraction of PCL in this PBC was 6.25% and it was named PBC-1. Similarly, PBCs with theoretical PCL mass fractions of 12.5%, 25%, and 37.5% were prepared by adjusting the PCL dosage, and were named PBC-2, PBC-3, and PBC-4, respectively. The actual mass fraction of PCL in PBC was determined by thermogravimetric analysis (TGA; Pyris 1 TGA, PerkinElmer, Waltham, MA, USA) [42].

### 3.3. Synthesis of nZVI@PBC

The nZVI@PBC composite was synthesized using the liquid-phase reduction method. A total of 4 g of PBC with an optimal mass ratio of PCL to BC (the optimization process is described in Section 2.5) was added into 150 mL of 20% (*v*/*v*) ethanol-water solution containing 24 g·L^−1^ FeCl_2_·4H_2_O. The mixture was vigorously stirred (250 rpm) at ambient temperature and purged with N_2_ for 30 min to remove dissolved oxygen. Subsequently, 150 mL of NaBH_4_ solution (35 g·L^−1^) was dropped into the above mixture using a peristaltic pump (NKCP-S10B, Kamoer, China) under an N_2_ atmosphere to allow the formation of nZVI with vigorous stirring at ambient temperature for 30 min. The reaction after the addition of the reducing solution can be described in Equation (1). The obtained samples were centrifuged at 9000 rpm for 10 min, and then rinsed several times with deionized water and anhydrous ethanol. Finally, the nZVI@PBC particles were dried in a vacuum oven (DZF6020, Liheng, China) at 50 °C, and stored in a vacuum drier for further use. The theoretical mass fraction of nZVI in this nZVI@PBC was 20% and it was named nZVI@PBC-20. Similarly, composites with theoretical nZVI mass fractions of 5%, 10%, and 30% were synthesized by adjusting the PBC dosage, and were named nZVI@PBC-5, nZVI@PBC-10 and nZVI@PBC-30, respectively. Inductively coupled plasma mass spectrometry (ICP-MS; iCAP Q, Thermo, MA, USA) was used to quantify the actual mass fraction of nZVI in the composite [43]. Pure nZVI particles were also synthesized under the same procedure without the PBC support.

Fe(II) + 2BH_4_^−^ + 6H_2_O → Fe(0) + 2BH(OH)_3_ + 7H_2_ (1)

### 3.4. Characterization

Some important properties of BC, PBC-2, and nZVI@PBC-20 were characterized. The surface and morphology of samples were observed by a scanning electron microscope (SEM, FEI Nova NanoSem 450, FEI, OR, USA). A transmission electron microscope (TEM, JEM-2100F, JEOL, Shizuoka, Japan) equipped with energy dispersive spectroscopy (EDS) was selected for further morphology and elemental distribution tests. The surface functional groups were characterized by a Fourier transform infrared spectroscope (FTIR, IS50, Thermo, MA, USA). The structures and crystal phases were investigated by an X-ray powder diffractometer (XRD, D8 ADVANCE Da Vinci, Bruker, Karlsruhe, Germany) using Cu-Kα radiation at 40 kV/30 mA.

### 3.5. Optimization of Mass Fractions

The optimal mass fraction of PCL in PBC was determined by characterizing the surface morphology and porous structures of PBCs with different PCL contents and evaluating their carbon-releasing characteristics in pure water. The surface morphology of samples was also observed using a SEM. The specific surface area and pore volume of the samples were analyzed by a Gemini VII2390 Brunauer-Emmett-Teller (BET) surface area analyzer (Micromeritics, GA, USA). The experiment to test carbon-releasing performance was conducted as follows: 0.8 g of PBCs with different mass ratios of PCL to BC were added into conical flasks with 80 mL of sterile deionized water. The flasks were then sealed with rubber stoppers and shaken for 360 h in a constant temperature oscillator (HNY-2102C, Honour, China) at 70 rpm and 25 °C. The well-mixed samples were taken periodically and centrifuged at 9000 rpm for 5 min. The resulting supernatants were filtered through 0.45 μm syringe filters and analyzed to determine the concentrations of total organic carbon (TOC) using a total organic carbon analyzer (multi NC 3100, Analytik Jena AG, Jena, Germany). After each sampling, equal sterile deionized water was supplemented in the conical flask. This experiment was conducted in triplicate, and the data are expressed as mean values with standard deviations.

The optimal mass fraction of nZVI in nZVI@PBC was determined by characterizing the porous structures of the composites with different nZVI contents and evaluating their synergy effects when coupled with CN32 on 1,1,1-TCA removal from groundwater. The specific surface area and pore volume were also analyzed by a BET surface area analyzer. Details of the 1,1,1-TCA removal experiment are described in Section 2.5.

### 3.6. Inoculation and Culture Condition

CN32 (ATCC 49138) was obtained from the China Centre of Industrial Culture Collection (Beijing, China). The strain was cultured aerobically in Luria-Bertani (LB) medium (tryptone 10 g/L, yeast extract 5.0 g/L, NaCl 10 g/L) at 30 °C for 12 h to reach the stationary phase [17]. The cells were harvested by centrifugation at 8500 rpm for 10 min, then washed three times with sterile PIPES buffer (20 mM, pH = 7). The obtained cells were then resuspended in a PIPES buffer solution to obtain a bacterial suspension. The cell concentration was determined by measuring the optical density at 600 nm (OD600) using a UV–Visible spectrophotometer (GENESYS 10S, Thermo, Waltham, MA, USA).

### 3.7. Batch Experiments

Batch experiments were conducted to explore the synergy effect of the composite and CN32 on the reductive dechlorination of 1,1,1-TCA. A certain amount of nZVI@PBC was added into a 100-mL serum bottle in a glove box filled with N_2_. Then, 78.4 mL of simulated groundwater with a certain concentration of 1,1,1-TCA (the preparation method was described previously [44] and 1.6 mL of bacterial suspension (OD600 = 1.0) were added. Subsequently, the bottles were capped with rubber stoppers and sealed with aluminium crimps. Afterward, all bottles were oscillated in a constant-temperature shaker (120 rpm, 30 °C) for 360 h. Samples were withdrawn at selected time intervals to analyze the 1,1,1-TCA concentration. The effects of nZVI mass fraction in the composite (5%, 10%, 20%, and 30%), nZVI@PBC dosage (0.25%, 0.50%, 1.00%, and 2.00%, *w/v*) and 1,1,1-TCA concentration (25, 50, 100 and 200 mg·L^−1^) on 1,1,1-TCA removal by nZVI@PBC + CN32 were investigated. Control experiments with CN32, PBC, nZVI, PBC + CN32, nZVI + CN32, and without any additions (Blank) in simulated groundwater were also conducted. Moreover, adsorbed Fe(II) and TOC concentrations, as well as bacterial abundance in the mixture, were measured to preliminarily discuss the improvement mechanism of 1,1,1-TCA removal by nZVI@PBC coupling with CN32. All experiments were conducted in triplicate, and the data are expressed as mean values with standard deviations.

### 3.8. Analysis Methods

At various time intervals, the mixture in each serum bottle was sampled and centrifuged at 9000 rpm for 5 min. Aliquots of the supernatant were analyzed for 1,1,1-TCA concentration by an automatic Purge and Trap Sample Concentrator (Teledyne Instruments, CA, USA) coupled to an Agilent 6890/5973 N gas chromatograph-mass spectrometer (P&TGC-MS). The concentration of total Fe(II) and dissolved Fe(II) in the supernatant were quantified using 1,10-phenanthroline at a wavelength of 512 nm by a spectrophotometer (GENESYS 10S, Thermo, MA, USA). The difference between the total Fe(II) and dissolved Fe(II) was defined as adsorbed Fe(II) [17]. The TOC concentration in the supernatant was measured with a TOC analyzer after N_2_ purging for 5 min to eliminate chlorinated hydrocarbons completely. The 16S rRNA gene copies of CN32 in the solid phase were quantified using a Real-Time PCR Detection System (iQ5, Bio-Rad, Hercules, CA, USA). The details of analysis methods mentioned above were described previously [45].

## 4. Conclusions and Implications

In this study, a novel composite of nZVI@PBC was successfully synthesized through the solution intercalation and liquid-phase reduction methods, and used for the removal of 1,1,1-TCA from the simulated groundwater in the present of *Shewanella putrefaciens* CN32. The results showed that PCL and Fe^0^ were uniformly distributed on the BC surface as irregular micro-particles and nanoparticles without obvious aggregation. The obtained composite had a relatively rough surface, being beneficial for the adsorption of pollutants and microorganism. It was found that nZVI@PBC coupling with CN32 could promote 1,1,1-TCA removal with high-efficiency and long-term performance. The component mass ratio had significant impacts on the surface morphology, carbon-releasing characteristics, and pollutant removal capacity of the composite, and the optimal mass ratio of PCL, BC, and nZVI was 1:7:2. The removal rate of 1,1,1-TCA increased with increasing composite dosage, but tended to be stable when exceeding 1.00% (*w*/*v*). Moreover, nZVI@PBC coupling with CN32 could effectively remove 1,1,1-TCA at various concentrations under optimal conditions. The mechanisms of 1,1,1-TCA removal enhanced by nZVI@PBC coupling with CN32 include the rapid chemical reductive dichlorination induced by nZVI in the early stage, and the microbial dissimilatory iron reduction in the remaining stage promoted by BC as colonization supporter and electron shuttle and PCL as slow-releasing carbon source.

In our study, the raw materials for the composite preparation are abundant, low cost, easy to obtain, and environmentally friendly. DIRB are widely distributed in both pristine and contaminated subsurface environments. Moreover, the above results demonstrated that the nZVI@PBC + CN32 system can not only effectively solve the aggregation and passivation problems of nZVI, but also prolong the reactivity of the composite. Therefore, it would have great potential practical application for the remediation of groundwater contaminated with chlorinated hydrocarbons.

## Figures and Tables

**Table 1:** Pore structure characteristics of nZVI@PBC.

Sample	Specific Surface Area (m^2^·g^−1^)	Total Pore Volume (cm^3^·g^−1^)	Micropore Volume (cm^3^·g^−1^)	Average Pore Diameter (nm)
nZVI@PBC-5	164.97	0.0637	0.0051	3.762
nZVI@PBC-10	172.33	0.0584	0.0049	3.917
nZVI@PBC-20	178.25	0.0561	0.0043	3.811
nZVI@PBC-30	146.39	0.0506	0.0040	3.542

## Data Availability

All data are presented in the text and in the Supplementary Materials. They are also available on request from the corresponding authors.

## Supplementary Materials

The following supporting information can be downloaded at: https://www.mdpi.com/article/10.3390/molecules28073145/s1. Figure S1: TEM images of nZVI@PBC-20 (a) and EDS with element mapping of nZVI@PBC-20 (b); Figure S2: SEM images of PBC-1 (a), PBC-2 (b), PBC-3 (c) and PBC-4 (d); Figure S3: FTIR spectra of nZVI@PBC-5, nZVI@PBC-10, nZVI@PBC-20 and nZVI@PBC-30; Figure S4: SEM images of nZVI@PBC-20 after 72 h of reaction; Table S1: Pore structure characteristics of BC and PBCs.
