# Supplementary material for: Polycaprolactone-Modified Biochar Supported Nanoscale Zero-Valent Iron Coupling with Shewanella putrefaciens CN32 for 1,1,1-Trichloroethane Removal from Simulated Groundwater: Synthesis, Optimization, and Mechanism
Source: Molecules. 2023 Mar 31;28(7):3145. doi: 10.3390/molecules28073145 (PMC10095663; doi:10.3390/molecules28073145)
Supplement: Supplementary file 1 [file molecules-28-03145-s001.zip › molecules-2248413-supplementary.pdf]

Supplementary materials - Polycaprolactone-Modified Biochar Supported Nanoscale Zero-Valent Iron Coupling with *Shewanella Putrefaciens* CN32 for 1,1,1- Trichloroethane Removal from Simulated Groundwater: Synthesis, Optimization, and Mechanism

Jing Ye [1], Yacen Mao [1], Liang Meng [2,3,4,5,*], Junjie Li [3,4], Xilin Li [3,4], Lishan Xiao [3,4], Ying Zhang [6], Fenghua Wang [7] and Huan Deng [8]

[1] School of Chemical and Environmental Engineering, Shanghai Institute of Technology, Shanghai 201418, China

[2] Key Lab of Eco-restoration of Regional Contaminated Environment, Ministry of Education, Shenyang University, Shenyang 110044, China

[3] School of Environmental and Geographical Sciences, Shanghai Normal University, Shanghai 200234, China

[4] Yangtze River Delta Urban Wetland Ecosystem National Field Scientific Observation and Research Station, Shanghai 201722, China

[5] State Environmental Protection Key Laboratory of Synergetic Control and Joint Remediation for Soil & Water Pollution, Chengdu University of Technology, Chengdu 610059, China

[6] The Shanghai Key Lab for Urban Ecological Processes and Eco-Restoration, East China Normal University, Shanghai 200241, China

[7] School of Geographical Sciences, Hebei Normal University, Shijiazhuang 050024, China

[8] School of Environment, Nanjing Normal University, Nanjing 210023, China

***** Correspondence: mengliang@shnu.edu.cn

Figure S1. TEM images of nZVI@PBC-20 (a) and EDS with element mapping of nZVI@PBC-20 (b).

Figure S2. SEM images of PBC-1 (a), PBC-2 (b), PBC-3 (c) and PBC-4 (d).

Figure S3. FTIR spectra of nZVI@PBC-5, nZVI@PBC-10, nZVI@PBC-20 and nZVI@PBC-30.

Figure S4. SEM images of nZVI@PBC-20 after 72 h of reaction.

Table S1. Pore structure characteristics of BC and PBCs.

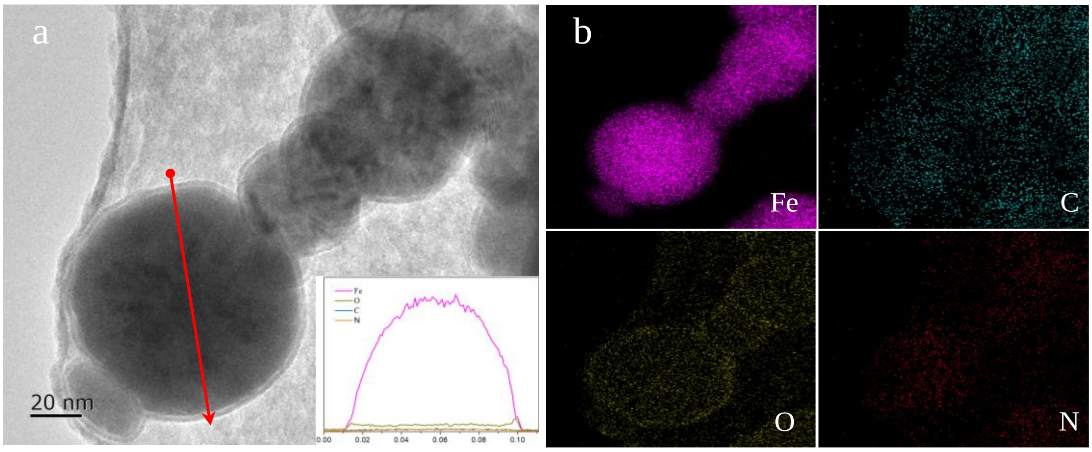

Figure S1. TEM images of nZVI@PBC-20 (a) and EDS with element mapping of nZVI@PBC-20 (b).

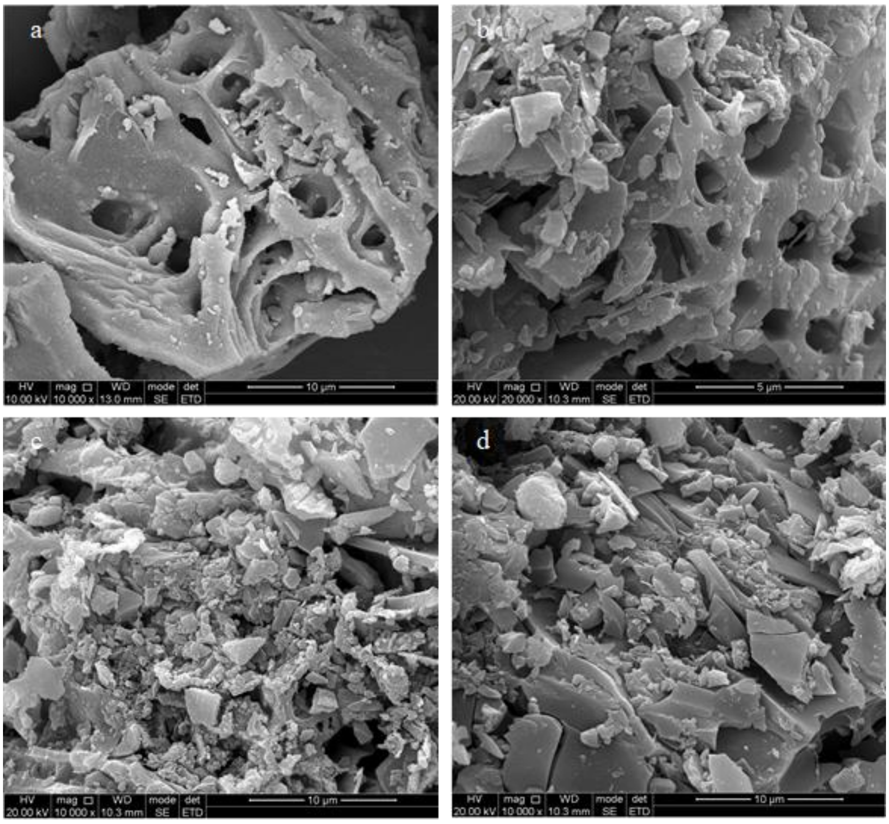

Figure S2. SEM images of PBC-1 (a), PBC-2 (b), PBC-3 (c) and PBC-4 (d).

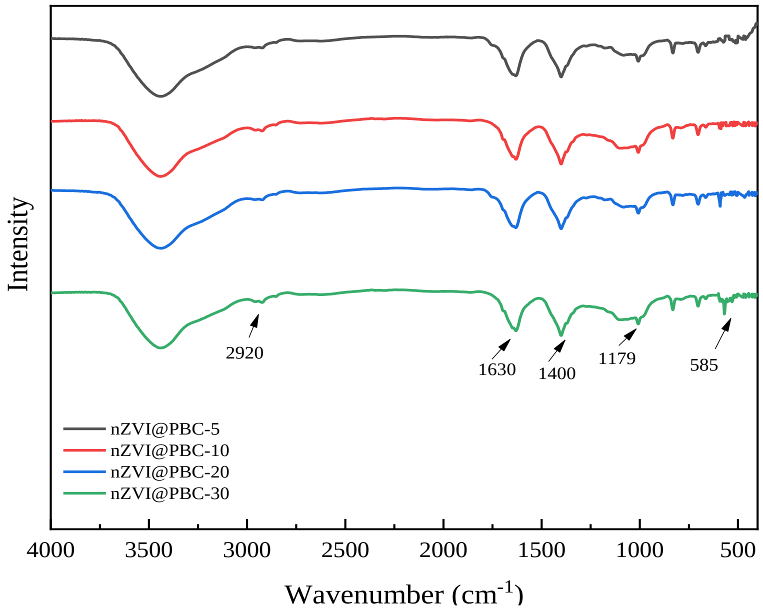

Figure S3. FTIR spectra of nZVI@PBC-5, nZVI@PBC-10, nZVI@PBC-20 and nZVI@PBC-30.

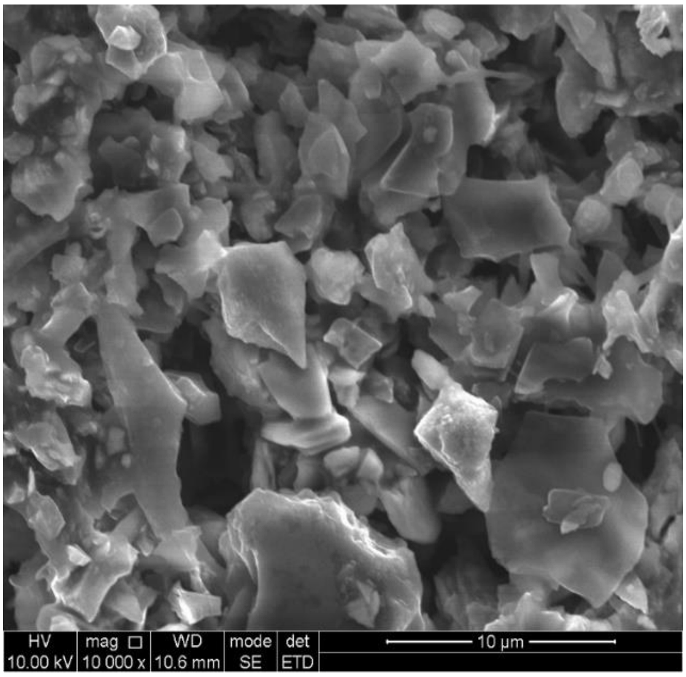

Figure S4. SEM images of nZVI@PBC-20 after 72 h of reaction.

Table S1. Pore structure characteristics of BC and PBCs.

Sample	Specific surface area ($m^2 \cdot g^{-1}$)	Total pore volume （$cm^3 \cdot g^{-1}$）	Micropore volume （$cm^3 \cdot g^{-1}$）	Average pore diameter （nm）
BC	196.35	0.1168	0.0490	3.943
PBC-1	179.71	0.0809	0.0053	3.862
PBC-2	158.96	0.0628	0.0050	3.716
PBC-3	112.32	0.0561	0.0047	2.517
PBC-4	90.67	0.0403	0.0051	2.943
